# Bridging the phenotype-target gap for molecular generation via multi-objective reinforcement learning

**DOI:** 10.1093/bioinformatics/btag242

**Published:** 2026-07-07

**Authors:** Haotian Guo, Hui Liu

**Affiliations:** College of Computer and Information Engineering, Nanjing Tech University, Nanjing, Jiangsu 211800, China; College of Computer and Information Engineering, Nanjing Tech University, Nanjing, Jiangsu 211800, China

## Abstract

**Motivation:**

The generation of high-quality candidate molecules remains a central challenge in AI-driven drug design. Current phenotype-based and target-based strategies each suffer limitations, either incurring high experimental costs or overlooking system-level cellular responses. To bridge this gap, we propose XMolRL, a novel generative framework that synergistically integrates phenotypic and target-specific cues for *de novo* molecular generation.

**Results:**

The phenotype-guided generator is first pretrained on expansive drug-induced transcriptional profiles and subsequently fine-tuned via multi-objective reinforcement learning (RL). Crucially, the reward function fuses docking affinity and drug-likeness scores, augmented with ranking loss, prior-likelihood regularization, and entropy maximization. The multi-objective RL steers the model toward chemotypes that are simultaneously potent, diverse, and aligned with the specified phenotypic effects. Extensive experiments demonstrate XMolRL’s superior performance over state-of-the-art phenotype-based and target-based models across multiple well-characterized targets. Our generated molecules exhibit favorable drug-like properties, high target affinity, and inhibitory potency (IC50) against cancer cells. This unified framework showcases the synergistic potential of combining phenotype-guided and target-aware strategies, offering a more effective solution for *de novo* drug discovery.

**Availability and implementation:**

The source code and datasets are available at: https://github.com/hliulab/XMolRL. The archived version of the source code and test data can be found at: https://doi.org/10.5281/zenodo.19607680.

## 1 Introduction

In AI-driven drug design, generating high-quality candidate molecules remains a fundamental challenge (Sanchez-Lengeling and Aspuru-Guzik 2018, Pang *et al.* 2023). In recent years, deep generative models have made significant advances in molecular design (Gómez-Bombarelli *et al.* 2018, Brown *et al.* 2019, Aini *et al.* 2024). Most existing methods largely fall into two distinct paradigms: target-based and phenotype-based drug discovery. Target-based approaches mainly focus on well-characterized targets—such as receptors, enzymes, and transport proteins (Imming *et al.* 2006, Hughes *et al.* 2011, Wishart *et al.* 2018)—and aim to design molecules that specifically bind to active sites or binding pockets. Target-driven approaches make advantage of protein structure or binding pocket information to generate molecules with high binding affinity (Danel *et al.* 2023). Representative autoregressive models, such as SBDD-3D (Luo *et al.* 2021) and Pocket2Mol (Peng *et al.* 2022), treat ligand generation as a stepwise 3D construction process. By encoding the binding pocket as geometric point clouds, these methods leverage rotationally invariant or equivariant networks to iteratively sample atoms and bonds, ensuring the generated structures strictly adhere to the specific binding site geometry. LiGAN (Méndez-Lucio *et al.* 2020) and AutoGrow4 (Spiegel and Durrant 2020) leverage the 3D geometry of protein binding pockets together with fragment-assembly heuristics to generate molecules that satisfy spatial docking constraints. By contrast, OptiMol (Nigam *et al.* 2021) and SampleDock (Xu *et al.* 2021) employ a “generate–evaluate–select” workflow: they sample candidates from a learned latent space, score each via molecular docking, retain the highest-scoring compounds, and iteratively refine the generator based on this feedback. Moreover, SBMolGen (Ma *et al.* 2021) integrates reinforcement learning by treating docking scores as rewards, thereby steering the generative policy toward progressively more favorable chemotypes. Nevertheless, drug efficacy is governed by the intricate cellular environment, and molecules optimized exclusively for target binding frequently fail to produce the intended phenotypic outcomes. These methods leverage structural information to guide rational drug design and elucidate mechanisms of action. Nevertheless, their effectiveness relies heavily on the availability of accurate target structures and well-understood biological functions (Swinney and Anthony 2011, Polykovskiy *et al.* 2020, von Lilienfeld *et al.* 2020). In addition, target-based methods often overlook system-level cellular responses, limiting their ability to capture off-target effects and broader phenotypic consequences.

In contrast, phenotype-based strategies seek to identify bioactive compounds that elicit desired phenotypic changes in cellular systems (Moffat *et al.* 2014, Vincent *et al.* 2022). This paradigm does not require prior knowledge of biological targets, allowing for a more holistic view of drug effects in complex cellular contexts. This is especially valuable when the underlying molecular mechanisms of a disease are poorly understood (Moffat *et al.* 2017, Meissner *et al.* 2022). However, large-scale experiments to observe drug-induced, system-level phenotypic effects remain prohibitively expensive and labor-intensive (Swinney and Anthony 2011). Also, these experiments are difficult to standardize or automate, limiting the throughput of phenotype-based drug screening (Moffat *et al.* 2017). Fortunately, transcriptional profiles effectively capture how compounds perturb the cellular state, offering molecular-level readouts that reflect drug perturbations leading to higher-order phenotypes (Lamb *et al.* 2006). Due to their high throughput and cost-efficiency, gene expression signatures have been widely used to probe both genetic alterations and external stimuli that lie along the causal pathway between genotype and disease phenotype. In fact, differential expression signatures have been employed to identify compounds capable of inducing desired phenotypic shifts (Subramanian *et al.* 2017). Phenotype-based molecular generation focuses on leveraging cellular-level response data—such as transcriptional profiles—to guide the design of compounds capable of modulating disease states (Moffat *et al.* 2014, Vincent *et al.* 2022). This approach is particularly valuable for complex or poorly understood diseases where molecular mechanisms remain elusive. With the integration of deep learning and omics technologies, phenotype-driven drug discovery models have seen rapid development. For example, PaccMann (Cadow *et al.* 2020) predicts drug sensitivity by modeling the relationship between gene expression profiles of cancer cell lines and compound SMILES representations, capturing phenotype-to-structure associations. TRIOMPHE (Kaitoh and Yamanishi 2021) employs a variational autoencoder conditioned on expression profiles to generate candidate molecules. Gex2SGen (Das *et al.* 2023) and GxVAEs (Li and Yamanishi 2024) followed similar ideas that used the desired expression profiles as input to design drug-like molecules capable of eliciting phenotypic changes. Méndez-Lucio *et al.* (2020) further employed a generative adversarial network (GAN) in conjunction with expression profiles to automatically design molecules with a high probability to induce therapeutic effects. SmilesGEN (Liu *et al.* 2025) is a dual-channel variational autoencoder framework that jointly models drug molecules and gene expression profiles in a shared latent space to generate drug-like molecules capable of eliciting desired phenotypic effects. Given the central role of transcriptional profiles in this study, we consistently use the term “phenotypic profile” to refer to transcriptional profiles.

To date, few efforts have leveraged the complementary strengths of target-based and phenotype-based paradigms. To bridge this gap, we propose XMolRL, a generative framework that integrates phenotypic and target-specific information for *de novo* molecular generation. XMolRL comprises two key components: (i) a phenotype-guided molecular generator trained on large-scale drug-induced phenotypic profiles enabling the conditional generation of molecules that elicit desired drug efficacy; and (ii) a reinforcement learning module that fine-tunes the generator using docking-based affinity scores to guide optimization toward specific protein targets. To ensure robust and diverse molecular generation, we incorporate multiple designed regularization terms into RL objective, to mitigate reward exploitation and maintain molecular diversity and drug-likeness. Our extensive experiments demonstrate that XMolRL can generate candidate molecules that simultaneously exhibit desired phenotypic effects, strong target affinity, and favorable drug-like properties. This unified framework showcases the synergistic potential of combining phenotype-driven and structure-aware strategies, offering a more comprehensive and effective solution for *de novo* drug discovery. Our main contributions are summarized as below: (i) We propose a novel framework that integrates phenotypic profiles with target protein structures to jointly optimize molecular efficacy and specificity. (ii) We introduce ranking loss, prior likelihood, and entropy regularization to enhance stability, diversity, and overall generation quality in reinforcement learning. (iii) Our method achieves superior performance over state-of-the-art phenotype-based and target-based models across multiple targets, simultaneously excelling in binding affinity, drug-likeness, and synthetic feasibility.

## 2 Materials and methods

### 2.1 XMolRL framework

**Figure 1:**
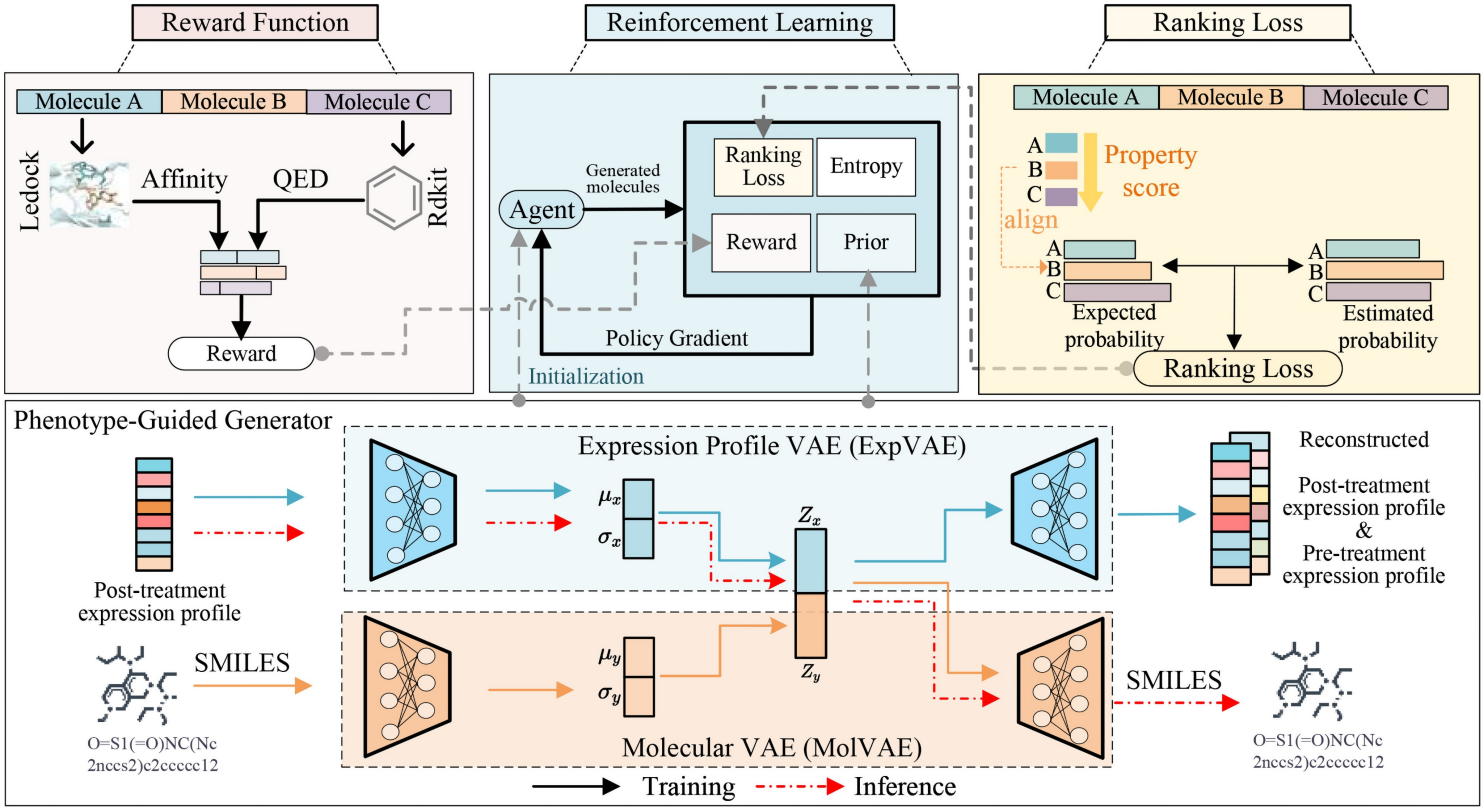
Overview of the XMolRL architecture. The model consists of a pretrained phenotypic-profile-guided generator, while a reinforcement learning agent fine-tunes the molecular generation toward desired biochemical properties.

We present XMolRL, a generative framework that integrates multi-modal data—including phenotypic profiles and chemical language—for the generation of drug-like molecules. As shown in Fig. 1, XMolRL comprises a phenotypic profile-guided generative module, and a reinforcement learning module that fine-tunes the agent toward desired molecular properties such as binding affinity and quantitative estimate of drug-likeness (QED) scores. The phenotypic generator is pretrained on large-scale drug-induced expression profiles and then acts as prior model. Next, RL is utilized to refine the agent model toward desired molecular attributes, while mitigating reward hacking effects that may compromise molecular uniqueness. Once trained, XMolRL generates molecules guided by expression profiles and informed by the structural context of target proteins.

### 2.2 Phenotype-guided generator

The phenotype-guided generator aims to produce drug-like molecules conditioned on desired transcriptional profiles. Inspired by SmilesGEN (Liu *et al.* 2025), we adopt a dual-channel variational autoencoder (VAE) architecture, which consists of ExpVAE, a multi-layer feedforward network used to encoding expression profiles, and MolVAE, a GRU-backboned VAE designed to encode and reconstruct molecular SMILES sequences. MolVAE is firstly pre-trained on a large-scale SMILES dataset, after which its encoder is frozen. Next, joint training is performed using triplets comprising SMILES sequences, perturbed expression profiles, and corresponding unperturbed profiles. This allows for the simultaneous reconstruction of both molecular structures and expression profiles, leading to joint optimization of the parameters in ExpVAE and MolVAE.

The dual-VAE architecture learns a latent space that captures the complex relationship between phenotypic signatures and chemical structures, enhancing its capacity to generate biologically relevant molecules. During inference, we retain the encoder from ExpVAE and the decoder from MolVAE to enable phenotype-to-molecule generation. The trained generator serves as a prior model in the RL phase, constraining the policy to produce molecules aligned with desired phenotypic effect.

### 2.3 Phenotype-target joint RL

To jointly optimize phenotypic efficacy and target binding, we cast phenotype and target-based molecular generation as a reinforcement learning (RL) problem. Specifically, the trained phenotypic generator serves as a prior, and an agent model adopts the same network architecture. During the RL phase, the agent is initialized with the prior’s parameters and optimized to maximize the task-specific reward function. Notably, the prior remains fixed throughout RL phase and serves as a regularizer, constraining the agent’s policy to generate molecules that induce biologically plausible phenotypic effects and preserving consistency with phenotype-conditioned distribution learned during pretraining.

To guide the generation of molecules with desirable properties, we incorporate both molecule-target docking scores and QED values into the reward function to encourage the agent to explore promising regions of the chemical space. The binding affinity between each generated molecule and its target protein is quantified using LeDock docking scores, a tool that has demonstrated strong performance in a recent benchmark study involving 2002 protein–ligand complexes (Zhao and Caflisch 2013, Wang *et al.* 2016). More precisely, the protein was first preprocessed using the LePro module, including hydrogen addition and desolvation. The docking pocket was defined based on the co-crystallized ligand, with the search space centered on the original ligand and extended by 10 Å along each boundary. Docking was then carried out using the default evolutionary algorithm settings in LeDock. For each molecule, 20 binding poses were generated, and the best docking score was used as the reward value. The QED score is computed using RDKit and incorporated into the reward function. Let $S=\{s_{1},s_{2},\ldots ,s_{n}\}$ denote a set of SMILES sequences, where each s∈S is of the form $s=\{a_{1},a_{2},\ldots ,a_{t}\}$, with *a* denoting a token in the sequence. For each SMILES sequence *s*, the reward function is defined as follows:


(1) Reward(s)=Dock(s)×QED(s)

where *Dock* denotes the normalized docking score and QED is the drug-likeness score of the generated molecule. A molecule is considered valid only if it satisfies RDKit’s validity check and its QED score exceeds a predefined threshold. We set the QED threshold to 0.34, which represents the mean score of compounds classified as “unattractive” or “too complex” (Bickerton *et al.* 2012). If a generated molecule is deemed invalid (e.g. chemically implausible or syntactically incorrect), its docking score is set to zero as a form of penalty. Moreover, we have observed that imposing a mild constraint on QED scores helps guide the generation process toward molecules with a more favorable balance of properties, preventing the reward function from being dominated solely by docking scores or QED values. To normalize the docking score into the range [0,1], we introduce a rescaling factor *k*, enabling better compatibility between different reward components. The full definition of the processed docking score is as follows:


(2) $$\mathrm{Dock}(s)=\{\begin{array}{c} \frac{\max\left(\mathrm{LeDock}(s),k\right)}{k},\;\text{if}\;s\;\text{is}\;\underline{\mathrm{valid}}\_\\ 0,\;\;\;\text{otherwise} \end{array}$$

The agent is trained to learn a policy π(θ) that maximizes the reward expectation, where θ represent the parameters of the agent model. Our approach builds upon the REINVENT framework (Loeffler *et al.* 2024), which has been shown effective in molecular generation tasks, with modifications tailored to our task. To improve training efficiency and stabilize gradient estimation, we adopt a mini-batch training strategy. At each step, a batch of *N* molecules is sampled from the agent. For a SMILES sequence *s* generated from $\pi_{\theta }$, we compute its log-likelihood $\text{log}\;p^{\left(\text{agent}\right)}(s)$ under the agent’s policy. We also obtain its prior log-likelihood $\text{log}\;p^{\left(\text{prior}\right)}(s)$ by passing the same sequence into the prior model. The objective of RL is to maximize both the prior log-likelihood and reward. Accordingly, we adopt the policy gradient algorithm to maximize the objective and thus the loss function is defined as follows:


(3) $$L_{\text{pg}}=-E_{S}[\text{log}\;p^{\left(\text{agent}\right)}(s;\theta )\cdot \mathrm{Reward}(s)]$$

### 2.4 Ranking loss and regularizers

Reinforcement learning in molecular generation often faces two key challenges: (i) sparse reward signals, which hinder effective policy learning, and (ii) unstable sample quality, where the agent may exploit the reward function by generating low-quality yet high-reward molecules. To mitigate these issues and encourage the model to assign higher generation probabilities to molecules with superior properties (e.g. binding affinity, QED, or biological activity), we introduce a ranking loss constraint. Let the desired property score of a molecule be denoted as AS(s), which is defined based on docking scores and relevant chemical properties. Given a pair of molecules $\left(s_{i},s_{j}\right)$ with $AS(s_{i})>AS(s_{j})$, we expect that the agent model assigns higher likelihood probability to the molecule with high property score. Specifically, the agent’s policy π is expected to satisfy the ranking constraint $p^{\left(\text{agent}\right)}(s_{i}|\pi (\theta ))>p^{\left(\text{agent}\right)}(s_{j}|\pi (\theta ))$ for all $AS(s_{i})>AS(s_{j})$. When a lower-quality molecule is assigned a higher generative probability than a higher-quality one, a ranking loss is incurred as below:


(4) $$\begin{array}{c} L_{\text{rank}}=\sum_{i}\sum_{j>i}\max\left(0,f(s_{j})-f(s_{i})+\gamma_{ij}\right),\\ \forall i<j,AS(s_{i})>AS(s_{j}) \end{array}$$

where $\gamma_{ij}=(j-i)\cdot \gamma$ is defined as the rank difference between two molecules multiplied by a hyperparameter γ. The function f(s) is defined as $\text{log}\;p^{\left(\text{agent}\right)}(s;\theta )$. By increasing the generative probability of high-quality molecules, RL no longer relies solely on sparse rewards, but instead incorporates fine-grained feedback through property-based ranking. This helps prevent the agent from overfitting to syntactically valid but chemically poor molecules. Compared to purely reward-driven optimization, the use of rank loss enables the model to effectively distinguish structurally similar molecules with significantly different properties, thereby enhancing its structural optimization capability.

To ensure the stability and effectiveness of RL, we introduce two additional regularizers: prior likelihood regularization (Gómez-Bombarelli *et al.* 2018, Loeffler *et al.* 2024) and entropy regularization (Zoph and Le 2017, You *et al.* 2018). To prevent the agent from deviating from the prior model, we incorporate a prior likelihood term based on the phenotype-guided generator. This regularizer is defined as:


(5) $$L_{\text{prior}}=-E_{S}[\text{log}\;p^{\left(\text{prior}\right)}(s)]$$

Here, $\text{log}\;p_{\text{prior}}(s)$ denotes the log-likelihood of a molecule *s* under the prior model. The prior poses the constraint that the generated molecules conform to valid SMILES syntax and chemically plausible structures, while also satisfying phenotype conditions with high fidelity. By imposing this prior constraint, the agent model benefits from the inductive biases encoded in the trained phenotypic generator, which improves its ability to respond accurately to phenotypic signatures. This is especially crucial in the early stages of RL, where the prior prevents the agent from cheating the reward function (“reward hacking”) by generating unrealistic or syntactically invalid molecules.

To encourage chemical space exploration and increase structural diversity, we introduce an entropy regularization term, which is defined as:


(6) $$L_{\text{ent}}=E_{S}\left[H(s;\theta )\right]$$

in which $H(s;\theta )=-\sum_{t=1}^{T}p(a|a_{<t};\theta )\cdot \;\text{log}\;p(a|a_{<t};\theta )$ denotes the entropy of the agent’s action distribution over the next token. Maximizing this entropy encourages stochasticity in the policy, helping the agent avoid deterministic local optima and promoting molecular diversity.

Taking the ranking loss and two regularization terms into the policy gradient loss, the overall objective enable jointly optimize molecular quality while preserving generative capacity. The final loss function is defined as:


(7) $$L=L_{\text{pg}}+\alpha \cdot L_{\text{rank}}+\beta \cdot L_{\text{prior}}-\lambda \cdot L_{\text{ent}}$$

where α, β, and λ are hyperparameters used to tradeoff the weights of the ranking loss, prior and entropy regularization, respectively. In this study, they are empirically tuned to 0.2, 0.8, and 0.1, respectively.

In our practice, we employed the bidirectional GRUs as the backbone of MolVAE. Both the encoder and decoder were composed of three hidden layers of size 192. The ExpVAE adopted multiple forward-feed layers with sizes 512, 256, and 192, respectively. During the training of phenotypic generator, the Adam optimizer is used with a learning rate of 5e-4 and a dropout rate of 0.1. The maximum length of the generated SMILES strings was restricted to 100 characters. In the RL stage, both agent models were optimized using Adam optimizer with a reduced learning rate of 1e-4. The batch size for both stages was set at 64. Our model was implemented using PyTorch 2.3.0. The training and all evaluation experiments were conducted on a CentOS Linux 8.2.2004 (Core) system, equipped with a GeForce RTX 4090 GPU and 128 GB of memory.

## 3 Experiments

### 3.1 Datasets and preprocessing

The MolVAE was pretrained using 10 032 879 SMILES sequences obtained from ZINC database (Irwin and Shoichet 2005), and then the ExpVAE and MolVAE were jointly trained using the L1000 dataset (Subramanian *et al.* 2017), which was a collective repository of transcriptional responses of human cell lines to drug exposures. We extracted the expression profiles of cell lines treated by 10 μM drug concentration for 24 h. To streamline the data, technical replicates were averaged. As a result, we obtained drug-induced expression profiles spanning 6549 drugs and 164 cell lines. The refined dataset comprised a total of 86 400 expression profiles across 978 landmark genes to fine-tune the dual VAEs. In addition, to evaluate the binding affinity of generated molecules toward target proteins, we collected co-crystal structures of the ten protein–ligand complexes from the RCSB Protein Data Bank. Protein structures were preprocessed using the LePro tool, and molecular docking was performed with the LeDock tool (http://www.lephar.com/). For performance evaluation, we used the expression profiles induced by the genetic perturbation of ten commonly target genes associated with the treatment of cancers. Among them, eight genes (AKT1, AKT2, AURKB, CTSK, EGFR, HDAC1, MTOR, and PIK3CA) were knockdown, and two genes (SMAD3 and TP53) were over-expressed. To evaluate the generated molecules, we obtained known ligands that have been confirmed to target these proteins from the Drug Target Commons (DTC) database (Tang *et al.* 2018). We compute the performance metrics between these known ligands and generated molecules.

### 3.2 Performance metrics

To evaluate the chemical properties of the generated molecules, we measured quite a few key metrics: validity, uniqueness, novelty, quantitative estimate of drug-likeness (QED), and synthetic accessibility (SA). High validity and uniqueness are indicators of an effective molecule generation process, while high novelty indicates the model avoids overfitting to the training set. For assessing binding affinity to target protein, we employed the docking scores computed by LeDock tool. In particular, we adopt half maximal inhibitory concentration (IC50) as a key performance metric for evaluating phenotype-guided generative models. IC50 represents the concentration of a compound required to inhibit 50% of cancer cell viability, with lower values indicating greater inhibitory potency. In our experiments, IC50 values were evaluated using the online PaccMann platform (https://ibm.biz/paccmann-aas) (Cadow *et al.* 2020). These performance metrics ensure that our model was comprehensively evaluated in generating molecules with potential therapeutic value and practical feasibility.

### 3.3 Comparison to phenotype-guided methods

We first compare XMolRL with several representative phenotype-based molecular generation methods, including SmilesGEN (Liu et al. 2025), GxVAEs (Li and Yamanishi 2024), and TRIOMPHE (Kaitoh and Yamanishi 2021). For each target protein, we generated 100 unique molecules using these methods and evaluated their performance across multiple metrics, as shown in Table 1. First, XMolRL achieved the best docking scores on 7 out of the 10 target proteins, with the generated molecules exhibiting significantly stronger binding affinities compared to those produced by SmilesGEN, GxVAEs, and TRIOMPHE. This highlights our model’s superior capability in optimizing chemical structure regarding specific binding pocket. Although GxVAEs performed comparably or slightly better on a few targets (e.g. AURKB and MTOR), its overall performance was inferior to our method, suggesting that our method offers better generalization and robustness across diverse protein contexts.

**Table 1:** Performance comparison of XMolRL versus phenotype-guided methods on affinity, QED and SA metrics.

	XMolRL			SmilesGEN			GxVAEs			TRIOMPHE		
	Affinity	QED	SA	Affinity	QED	SA	Affinity	QED	SA	Affinity	QED	SA
AKT1	**−6.49**	**0.764**	**2.634**	−5.23	0.585	2.932	−5.77	0.583	3.207	−2.33	0.462	4.08
AKT2	**−6.34**	**0.729**	**2.844**	−5.84	0.610	3.06	−6.16	0.595	3.42	−2.95	0.445	4.25
AURKB	−6.71	**0.736**	**2.612**	−6.21	0.574	3.01	**−6.94**	0.599	3.276	−2.31	0.425	4.10
CTSK	−5.07	**0.675**	3.00	−4.81	0.604	**2.97**	**−5.33**	0.597	3.011	−1.87	0.399	4.47
HDAC1	**−4.64**	**0.729**	**3.197**	−3.51	0.566	3.017	−3.59	0.619	3.225	−1.84	0.437	4.30
MTOR	−6.27	**0.730**	**2.685**	−5.96	0.57	3.00	**−6.67**	0.605	3.324	−2.75	0.465	4.18
PIK3CA	**−6.04**	**0.731**	3.132	−5.65	0.578	**2.98**	−5.86	0.640	3.298	−2.88	0.435	4.50
SMAD3	**−5.16**	**0.676**	**3.212**	−3.54	0.577	3.01	−3.4	0.594	3.242	−2.57	0.464	4.15
TP53	**−7.01**	**0.755**	**2.81**	−4.56	0.593	2.97	−4.38	0.611	3.281	−2.64	0.417	4.17
EGFR	**−7.37**	**0.726**	**2.464**	−6.67	0.57	2.95	−7.33	0.58	3.58	−3.31	0.442	4.37

Bold values indicate the best performance for each metric.

Moreover, XMolRL consistently achieved the highest QED scores across all ten target proteins, indicating that the generated molecules exhibit more drug-like pharmacological properties. Notably, even though SA scores were not explicitly included in the RL reward function, XMolRL still outperformed the competing methods on most targets with respect to SA metric. We attribute the superior SA performance of XMolRL to two factors. First, the pretrained generative model captures the distribution of chemically reasonable structures from large-scale SMILES data, promoting better synthetic accessibility. Second, the reinforcement learning stage jointly optimizes multiple reward functions, implicitly enforcing chemical validity and structural reasonableness, further enhancing synthesizability. We further analyzed the distribution of molecular property metrics across different methods. As shown in Fig. S1, available as supplementary data at *Bioinformatics* online, XMolRL achieved substantially higher affinity than TRIOMPHE and SmilesGEN, with peaks concentrated in the high-affinity region (lower score means higher affinity). Meanwhile, XMolRL consistently outperformed all competing methods in QED and SA score distributions, demonstrating its superior ability to generate drug-like and synthetically accessible compounds. To further validate the structural fidelity, we calculated the maximum Tanimoto similarity between the generated molecules and known ligands for each target. As illustrated in Fig. S2, available as supplementary data at *Bioinformatics* online, the generated molecules achieving the highest similarity scores are displayed alongside their corresponding reference ligands, verifying XMolRL’s capability to accurately reproduce key structural features of active compounds.

**Figure 2:**
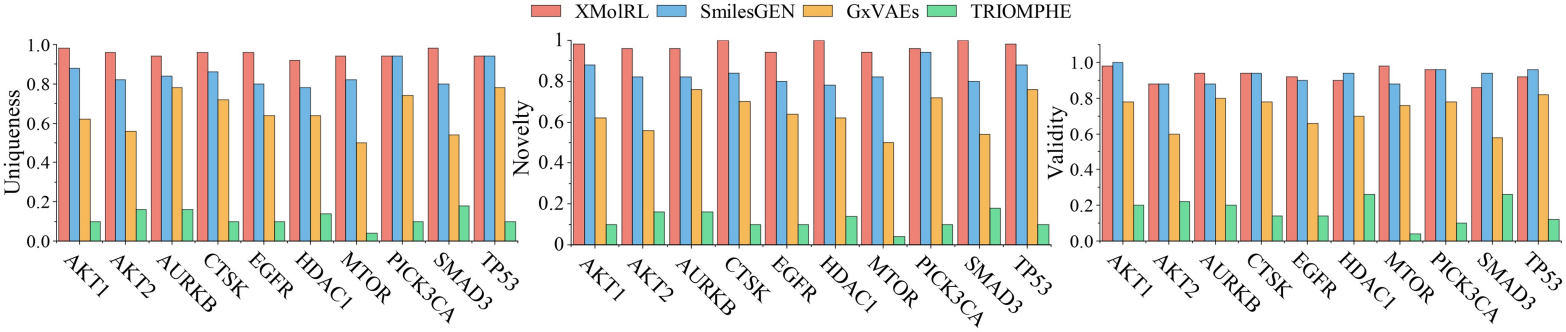
Performance comparison of XMolRL to three representative phenotype-guided methods on uniqueness, novelty, and validity.

Moreover, we evaluated the uniqueness, novelty, and validity of our generated molecules. As shown in Fig. 2, XMolRL outperformed four competing methods on the vast majority of targets, with novelty reaching 100% on targets such as TP53. These results demonstrate that XMolRL consistently generates structurally novel compounds unseen during training.

### 3.4 Comparison to target-based methods

**Figure 3:**
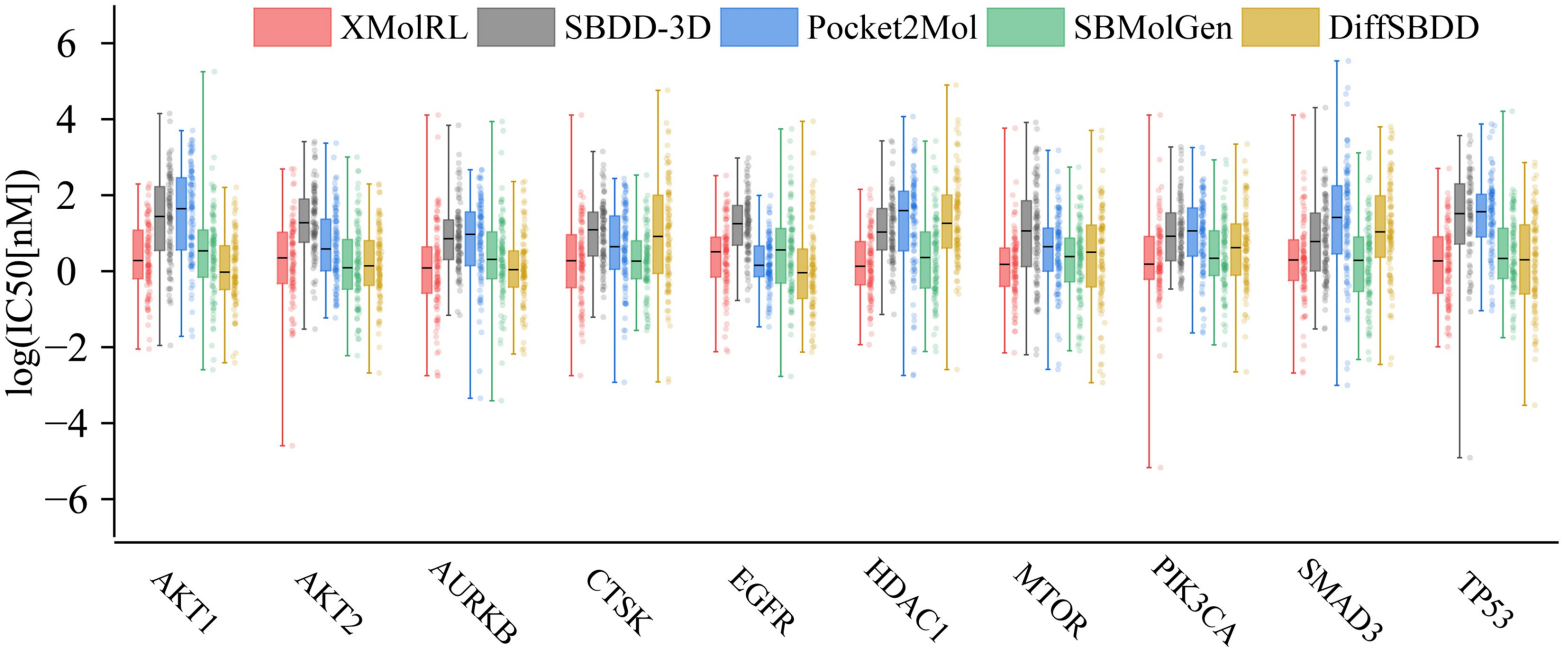
Docking confirmation of generated molecules binding to corresponding target proteins, as well as molecular properties and binding affinity.

We next compared XMolRL with four docking-based generative methods: SBDD-3D (Luo *et al.* 2021), Pocket2Mol (Peng *et al.* 2022), SBMolGen (Ma *et al.* 2021), and DiffSBDD (Schneuing *et al.* 2024). Similarly, we generated 100 unique molecules using each method for performance evaluation. As shown in Table 2, XMolRL outperformed the competing methods in both docking scores and QED. We used EGFR as a representative target to analyze the distribution of evaluation metrics. As shown in Fig. S3, available as supplementary data at *Bioinformatics* online, XMolRL exhibited substantial advantage over other methods in both docking and QED scores over competing methods, with peaks concentrated in the advantageous region. These results demonstrate that XMolRL effectively leveraged both phenotypic guidance and target-specific docking signals, leading to superior molecular generation performance across multiple evaluation metrics. Furthermore, Fig. 3 visualized the docking poses of the top-performing candidate molecules for each target, which indicate that the generated ligands adopt stable and reasonable conformations within the active sites and form effective interactions with key residues.

**Table 2:** Performance comparison of XMolRL versus four representative target-based methods on affinity, QED and SA metrics.

	XMolRL			Pocket2Mol			SBDD-3D			SBMolGen			DiffSBDD		
	Affinity	QED	SA	Affinity	QED	SA	Affinity	QED	SA	Affinity	QED	SA	Affinity	QED	SA
AKT1	−6.49	**0.764**	**2.634**	−2.82	0.436	2.622	−2.82	0.525	3.26	−4.76	0.722	2.897	**−6.56**	0.417	4.749
AKT2	−6.34	**0.729**	**2.844**	−5.99	0.612	3.362	**−6.99**	0.399	5.42	−5.49	0.710	3.042	−6.43	0.469	4.957
AURKB	−6.71	0.736	2.612	−5.47	0.687	**2.600**	−6.83	0.566	5.36	−5.91	**0.737**	2.927	**−6.84**	0.445	4.673
CTSK	**−5.07**	0.675	**3.00**	−4.67	**0.723**	2.803	−4.55	0.672	4.98	−4.61	0.713	3.032	−3.80	0.463	3.536
HDAC1	**−4.64**	**0.729**	3.197	−2.35	0.507	**2.535**	−2.83	0.618	2.86	−3.54	0.713	2.95	−2.62	0.502	3.568
MTOR	**−6.27**	**0.730**	**2.685**	−5.68	0.639	3.189	−5.22	0.559	5.748	−5.43	0.727	2.970	−5.41	0.487	4.403
PIK3CA	**−6.04**	0.731	3.132	−4.70	**0.750**	**2.826**	−4.47	0.612	4.25	−5.53	0.706	2.936	−5.50	0.612	4.349
SMAD3	**−5.16**	0.676	3.212	−3.72	0.598	**2.459**	−3.31	0.385	3.91	−3.92	**0.747**	3.091	−3.89	0.519	3.466
TP53	**−7.01**	**0.755**	**2.81**	−5.25	0.656	3.351	−5.16	0.573	5.127	−5.51	0.709	2.992	−4.63	0.442	4.968
EGFR	**−7.37**	0.726	**2.464**	−7.02	0.610	3.469	−6.32	0.566	5.68	−6.14	**0.737**	3.062	−6.27	0.478	4.844

Bold values indicate the best performance for each metric.

**Figure 4:**
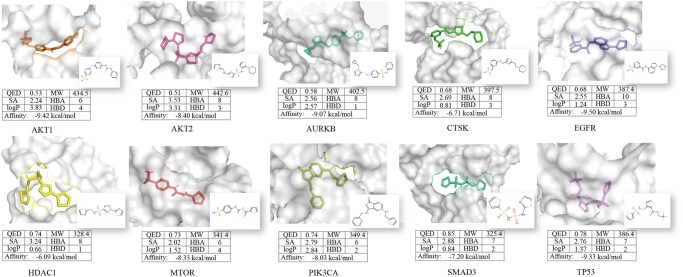
Performance comparison of XMolRL versus four representative targeted approaches in suppressing cancer cell viability.

Target-based methods focus solely on generating high-affinity molecules to specific target proteins, overlooking the effect on cellular contexts. In contrast, XMolRL explicitly conditioned on the cellular state of the target protein to generate molecules that would induce desirable phenotypic profiles. To assess the effectiveness of this strategy, we further evaluated the ability of generated molecules to inhibit the viability of MCF7 breast cancer cells. Fig. 4 presents boxplots comparing the predicted drug sensitivity—expressed as the logarithm of IC50 values estimated by PaccMann (Cadow *et al.* 2020)—of molecules generated by XMolRL and baseline methods across 10 target proteins. These metrics serve as a quantitative measure of the generated molecules’ inhibitory potency against cancer cells. Note that lower IC50 values indicate stronger inhibitory capacity. It can be observed that the sensitivity of XMolRL-generated molecules exhibited relatively lower mean and variance, indicating its stability to generate high-quality molecules. While SBMolGen did generate a few highly potent molecules with extremely low sensitivity values for specific targets such as AKT2 and SMAD, its overall distribution was broader and less stable. The results verified that XMolRL produced molecules with stronger or comparable anticancer potential compared to four comparative methods. To further validate these observations, we performed pairwise comparisons between XMolRL and other target-based molecular generation methods using the Wilcoxon rank-sum test. To control for the inflation of false positives caused by multiple comparisons, we further applied multiple testing correction to the resulting *P*-values. Moreover, we reported Cliff’s delta as an effect size metric to assess both the magnitude and direction of the differences between methods (Table S1, available as supplementary data at *Bioinformatics* online). The results indicate that XMolRL has significant advantages over SBDD-3D across nearly all targets, and shows significant benefits for several targets compared to Pocket2Mol and DiffSBDD. By contrast, the comparison with SBMolGen does not reveal overall significant differences, suggesting that our method achieves comparable performance to SBMolGen. These results highlight that XMolRL’s phenotype-conditioned strategy enables more effective and personalized molecule design, particularly for targeting cancer-specific cellular states.

### 3.5 Therapeutic molecular generation

**Figure 5:**
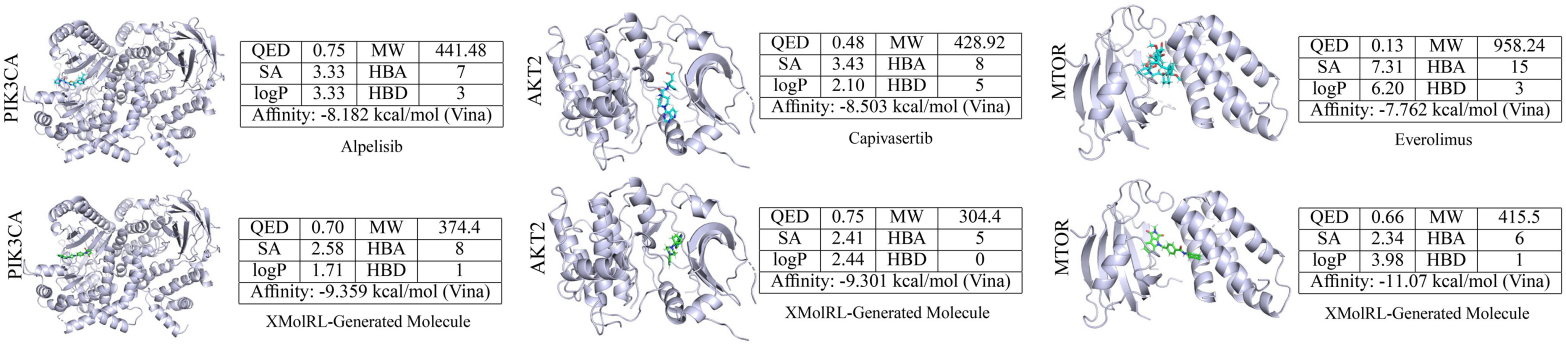
Comparison of XMolRL-generated molecules versus approved drugs biding to PIK3CA, AKT2, and mTOR targets, respectively.

To assess XMolRL’s ability to generate therapeutic compounds, we produced 100 novel molecules conditioned on breast cancer expression profiles and targeting PIK3CA, AKT2, and MTOR. These molecules were benchmarked against three FDA-approved drugs: Alpelisib (PI3Kα inhibitor), Capivasertib (AKT inhibitor), and Everolimus (mTOR inhibitor). All candidates were firstly filtered according to Lipinski’s Rule (LogP < 5, MW < 500 Da, H-bond donors < 5, H-bond acceptors < 10), and then were docked to the target proteins using AutoDock Vina (Trott and Olson 2010). Other key physicochemical properties, including QED and synthetic accessibility (SA), were also computed for further evaluation. As shown in Fig. 5, we illustrate the docking conformation of the three approved drugs (upper) and XMolRL-generated candidates (bottom), alongside their QED, SA, and Vina scores. XMolRL-generated molecules consistently outperformed the known drugs in binding affinity, achieving Vina scores of −9.359, −9.301, and −11.070 kcal/mol versus −8.182, −8.503, and −7.762 kcal/mol for Alpelisib, Capivasertib, and Everolimus, respectively. These candidates also exhibited improved drug-likeness and SA scores. Taking mTOR as example, Everolimus scored a QED of only 0.13 with an SA score of 7.31, whereas XMolRL-generated candidate achieved a QED of 0.66 and an SA score of 2.34. Moreover, the generated candidate’s molecular weight (415.5 Da) and Log *P* align more closely with Lipinski criteria than Everolimus (958.2 Da), suggesting enhanced absorption and bioavailability. These results demonstrate that XMolRL not only generates novel compounds with superior target binding, but also optimizes critical physicochemical properties.

### 3.6 Ablation study

To evaluate the contribution of key components in XMolRL, we conducted ablation studies on the EGFR target by removing the ranking loss or replacing multi-objective optimization with single-objective optimization (Affinity or QED) (Table 3). The results show that the ranking loss is crucial for maintaining molecular uniqueness: its removal reduces uniqueness from 96% to 56%, indicating severe mode collapse and metric inflation due to repeated sampling. Single-objective optimization also exhibits clear bias—optimizing affinity alone degrades QED and synthetic accessibility (SA), while optimizing QED alone substantially compromises binding affinity. In contrast, the full XMolRL model effectively balances diversity, drug-likeness, and synthetic feasibility without sacrificing docking performance, resulting in more practically valuable candidate molecules. To further assess the generalization ability of our model, we also conducted ablation experiments on three other targets, AKT1, TP53, and mTOR, with the results reported in Tables S2–S4, available as supplementary data at *Bioinformatics* online, respectively. These results demonstrate that our method achieves consistent performance improvements across multiple targets, confirming that the effectiveness of each component is not specific to any single target.

**Table 3:** Performance metrics achieved by ablated models.

Metric	XMolRL	w/o Rank	w/o QED	w/o Affinity
Uniqueness (%)	96	56	75	98
Validity (%)	100	98	96	90
Novelty (%)	94	96	100	98
Affinity (↓)	−7.53	−6.89	−7.54	−5.35
QED (↑)	0.717	0.748	0.504	0.770
SA (↓)	2.54	2.63	3.01	2.72

## 4 Conclusion

In this study, we present XMolRL, a deep learning–based molecular generation framework that explicitly integrates phenotypic profile with target-specific constraints. Unlike existing approaches that rely solely on either cellular phenotypes or protein structures, XMolRL unifies these two complementary paradigms within a single learning framework. By conditioning molecular generation on drug-induced transcriptional profiles and further optimizing candidates using target-aware reinforcement learning, the proposed method enables the generation of molecules that are not only structurally compatible with specific protein targets but also aligned with desired cellular-level phenotypic effects. This joint modeling strategy provides a more biologically meaningful objective for *de novo* molecular design, particularly in complex disease contexts where phenotypic responses cannot be fully explained by target binding alone. Moreover, the introduction of ranking-based optimization and regularization terms improves training stability and mitigates common issues such as reward hacking and mode collapse, leading to enhanced molecular diversity and drug-likeness. These design choices highlight the potential of deep learning models to capture cross-scale relationships between molecular structure, target interaction, and cellular response.

Despite these advantages, XMolRL has several limitations. First, the framework relies on docking-based affinity estimation, which provides only an approximate measure of protein–ligand binding. The limited accuracy of such predictors also introduces noise into the optimization process and affects the quality of generated molecules. Second, accurate prediction of drug sensitivity for newly designed molecules remains a major challenge. Current predictive models are not sufficiently reliable to estimate induced cell death for unseen compounds. As a result, phenotypic objectives cannot yet be seamlessly incorporated as explicit reward signals within the reinforcement learning framework.

In summary, we propose a deep learning framework that jointly integrates phenotypic profiles and target-specific information for *de novo* molecular generation. By unifying cellular-level phenotypic guidance with target-aware optimization, XMolRL enables the generation of biologically relevant, drug-like molecules and provides a promising direction for phenotype–target integrated drug discovery.

## Supplementary Material

btag242_Supplementary_Data

## Data Availability

The source code and datasets for the XMolRL framework are available in our GitHub repository at https://github.com/hliulab/XMolRL. The archived version of the source code, datasets, and test data is available on Zenodo at https://doi.org/10.5281/zenodo.19607680. The molecular SMILES set used for pretraining was obtained from the ZINC database (https://zinc.docking.org/). The transcriptional response profiles used for joint training and fine-tuning were sourced from the L1000 project (https://clue.io/data/CMap2020#LINCS2020). Protein co-crystal structures for docking evaluations were collected from the RCSB Protein Data Bank (PDB) (https://www.rcsb.org/). Known reference ligands used for benchmarking were obtained from the ChEMBL (https://www.ebi.ac.uk/chembl/). Predicted drug sensitivity values were estimated using the PaccMann (https://paccmann.github.io/). Any additional information required to re-analyze the data reported in this paper is available from the corresponding author upon request.

## References

[btag242-B1] Aini NS , Ansori ANM, Herdiansyah MA et al Antimalarial potential of phytochemical compounds from *Garcinia atroviridis* griff ex. T. anders targeting multiple proteins of *Plasmodium falciparum* 3D7: an in silico approach. BIO Integration 2024;5:967.

[btag242-B2] Bickerton GR , Paolini GV, Besnard J et al Quantifying the chemical beauty of drugs. Nat Chem 2012;4:90–8. 22270643 10.1038/nchem.1243 PMC3524573

[btag242-B3] Brown N , Fiscato M, Segler MH et al GuacaMol: benchmarking models for de novo molecular design. J Chem Inf Model 2019;59:1096–108. 30887799 10.1021/acs.jcim.8b00839

[btag242-B4] Cadow J , Born J, Manica M et al PaccMann: a web service for interpretable anticancer compound sensitivity prediction. Nucleic Acids Res 2020;48:W502–8. 32402082 10.1093/nar/gkaa327 PMC7319576

[btag242-B5] Danel T , Łęski J, Podlewska S et al Docking-based generative approaches in the search for new drug candidates. Drug Discov Today 2023;28:103439. 36372330 10.1016/j.drudis.2022.103439

[btag242-B6] Das D , Chakrabarty B, Srinivasan R et al Gex2SGen: designing drug-like molecules from desired gene expression signatures. J Chem Inf Model 2023;63:1882–93. 36971750 10.1021/acs.jcim.2c01301

[btag242-B7] Gómez-Bombarelli R , Wei JN, Duvenaud D et al Automatic chemical design using a data-driven continuous representation of molecules. ACS Cent Sci 2018;4:268–76. 29532027 10.1021/acscentsci.7b00572 PMC5833007

[btag242-B8] Hughes JP , Rees S, Kalindjian SB et al Principles of early drug discovery. Br J Pharmacol 2011;162:1239–49. 21091654 10.1111/j.1476-5381.2010.01127.x PMC3058157

[btag242-B9] Imming P , Sinning C, Meyer A. Drugs, their targets and the nature and number of drug targets. Nat Rev Drug Discov 2006;5:821–34. 17016423 10.1038/nrd2132

[btag242-B10] Irwin JJ , Shoichet BK. Zinc—a free database of commercially available compounds for virtual screening. J Chem Inf Model 2005;45:177–82. 15667143 10.1021/ci049714 PMC1360656

[btag242-B11] Kaitoh K , Yamanishi Y. TRIOMPHE: transcriptome-based inference and generation of molecules with desired phenotypes by machine learning. J Chem Inf Model 2021;61:4303–20. 34528432 10.1021/acs.jcim.1c00967

[btag242-B12] Lamb J , Crawford ED, Peck D et al The connectivity map: using gene-expression signatures to connect small molecules, genes, and disease. Science (1979) 2006;313:1929–35. 10.1126/science.1132939 17008526

[btag242-B13] Li C , Yamanishi Y. GxVAEs: two joint vaes generate hit molecules from gene expression profiles. In: *Proceedings of the AAAI Conference on Artificial Intelligence*, Vol. 38. Vancouver, Canada: AAAI Press, 2024, 13455–63.

[btag242-B14] Liu H , Tian S, Liu X. Phenotypic profile-informed generation of drug-like molecules via dual-channel variational autoencoders. In: Kwok J (ed.), *Proceedings of the Thirty-Fourth International Joint Conference on Artificial Intelligence (IJCAI-25),* 2025, 9068–76.

[btag242-B15] Loeffler HH , He J, Tibo A et al Reinvent 4: modern AI-driven generative molecule design. J Cheminform 2024;16:20. 38383444 10.1186/s13321-024-00812-5 PMC10882833

[btag242-B16] Luo S , Guan J, Ma J et al A 3D generative model for structure-based drug design. Adv Neural Inf Process Syst 2021;34:6229–39.

[btag242-B17] Ma B , Terayama K, Matsumoto S et al Structure-based de novo molecular generator combined with artificial intelligence and docking simulations. J Chem Inf Model 2021;61:3304–13. 34242036 10.1021/acs.jcim.1c00679

[btag242-B18] Meissner F , Geddes-McAlister J, Mann M et al The emerging role of mass spectrometry-based proteomics in drug discovery. Nat Rev Drug Discov 2022;21:637–54. 35351998 10.1038/s41573-022-00409-3

[btag242-B19] Méndez-Lucio O , Baillif B, Clevert D-A et al De novo generation of hit-like molecules from gene expression signatures using artificial intelligence. Nat Commun 2020;11:10. 31900408 10.1038/s41467-019-13807-w PMC6941972

[btag242-B20] Moffat JG , Rudolph J, Bailey D. Phenotypic screening in cancer drug discovery—past, present and future. Nat Rev Drug Discov 2014;13:588–602. 25033736 10.1038/nrd4366

[btag242-B21] Moffat JG , Vincent F, Lee JA et al Opportunities and challenges in phenotypic drug discovery: an industry perspective. Nat Rev Drug Discov 2017;16:531–43. 28685762 10.1038/nrd.2017.111

[btag242-B22] Nigam A , Pollice R, Krenn M et al Beyond generative models: superfast traversal, optimization, novelty, exploration and discovery (stoned) algorithm for molecules using selfies. Chem Sci 2021;12:7079–90. 34123336 10.1039/d1sc00231g PMC8153210

[btag242-B23] Pang C , Qiao J, Zeng X et al Deep generative models in de novo drug molecule generation. J Chem Inf Model 2023;64:2174–94. 37934070 10.1021/acs.jcim.3c01496

[btag242-B24] Peng X , Luo S, Guan J et al Pocket2mol: efficient molecular sampling based on 3D protein pockets. In: Chaudhuri K, Jegelka S, Song L et al. (eds), *Proceedings of the 39th International Conference on Machine Learning*, Baltimore, Maryland, USA: PMLR, 2022, 17644–55.

[btag242-B25] Polykovskiy D , Zhebrak A, Sanchez-Lengeling B et al Molecular sets (MOSES): a benchmarking platform for molecular generation models. Front Pharmacol 2020;11:565644. 33390943 10.3389/fphar.2020.565644 PMC7775580

[btag242-B26] Sanchez-Lengeling B , Aspuru-Guzik A. Inverse molecular design using machine learning: generative models for matter engineering. Science 2018;361:360–5. 30049875 10.1126/science.aat2663

[btag242-B27] Schneuing A , Harris C, Du Y et al Structure-based drug design with equivariant diffusion models. Nat Comput Sci 2024;4:899–909. 39653846 10.1038/s43588-024-00737-x PMC11659159

[btag242-B28] Spiegel JO , Durrant JD. AutoGrow4: an open-source genetic algorithm for de novo drug design and lead optimization. J Cheminform 2020;12:25. 33431021 10.1186/s13321-020-00429-4 PMC7165399

[btag242-B29] Subramanian A , Narayan R, Corsello SM et al A next generation connectivity map: L1000 platform and the first 1,000,000 profiles. Cell 2017;171:1437–52.e17. 29195078 10.1016/j.cell.2017.10.049 PMC5990023

[btag242-B30] Swinney DC , Anthony J. How were new medicines discovered? Nat Rev Drug Discov 2011;10:507–19. 21701501 10.1038/nrd3480

[btag242-B31] Tang J , Tanoli Z-U-R, Ravikumar B et al Drug target commons: a community effort to build a consensus knowledge base for drug-target interactions. Cell Chem Biol 2018;25:224–9.e2. 29276046 10.1016/j.chembiol.2017.11.009 PMC5814751

[btag242-B32] Trott O , Olson AJ. AutoDock Vina: improving the speed and accuracy of docking with a new scoring function, efficient optimization, and multithreading. J Comput Chem 2010;31:455–61. 19499576 10.1002/jcc.21334 PMC3041641

[btag242-B33] Vincent F , Nueda A, Lee J et al Phenotypic drug discovery: recent successes, lessons learned and new directions. Nat Rev Drug Discov 2022;21:899–914. 35637317 10.1038/s41573-022-00472-w PMC9708951

[btag242-B34] von Lilienfeld OA , Müller K-R, Tkatchenko A. Exploring chemical compound space with quantum-based machine learning. Nat Rev Chem 2020;4:347–58. 37127950 10.1038/s41570-020-0189-9

[btag242-B35] Wang Z , Sun H, Yao X et al Comprehensive evaluation of ten docking programs on a diverse set of protein–ligand complexes: the prediction accuracy of sampling power and scoring power. Phys Chem Phys 2016;18:12964–75. 10.1039/c6cp01555g 27108770

[btag242-B36] Wishart DS , Feunang YD, Guo AC et al DrugBank 5.0: a major update to the DrugBank database for 2018. Nucleic Acids Res 2018;46:D1074–82. 29126136 10.1093/nar/gkx1037 PMC5753335

[btag242-B37] Xu Z , Wauchope OR, Frank AT. Navigating chemical space by interfacing generative artificial intelligence and molecular docking. J Chem Inf Model 2021;61:5589–600. 34633194 10.1021/acs.jcim.1c00746

[btag242-B38] You J , Liu B, Ying Z et al Graph convolutional policy network for goal-directed molecular graph generation. Adv Neural Inf Process Syst 2018;31:6410–21.

[btag242-B39] Zhao H , Caflisch A. Discovery of ZAP70 inhibitors by high-throughput docking into a conformation of its kinase domain generated by molecular dynamics. Bioorg Med Chem Lett 2013;23:5721–6. 23993776 10.1016/j.bmcl.2013.08.009

[btag242-B40] Zoph B , Le QV. Neural architecture search with reinforcement learning. In: *5th International Conference on Learning Representations (ICLR 2017)*. Toulon, France, 2017.

