# Supplementary material for: Bridging the phenotype-target gap for molecular generation via multi-objective reinforcement learning
Source: Bioinformatics. 2026 Jul 7;42(Suppl 1):btag242. doi: 10.1093/bioinformatics/btag242 (PMC13340265; doi:10.1093/bioinformatics/btag242)
Supplement: btag242_Supplementary_Data [file btag242_supplementary_data.pdf]

Supplementary file of XMolRL

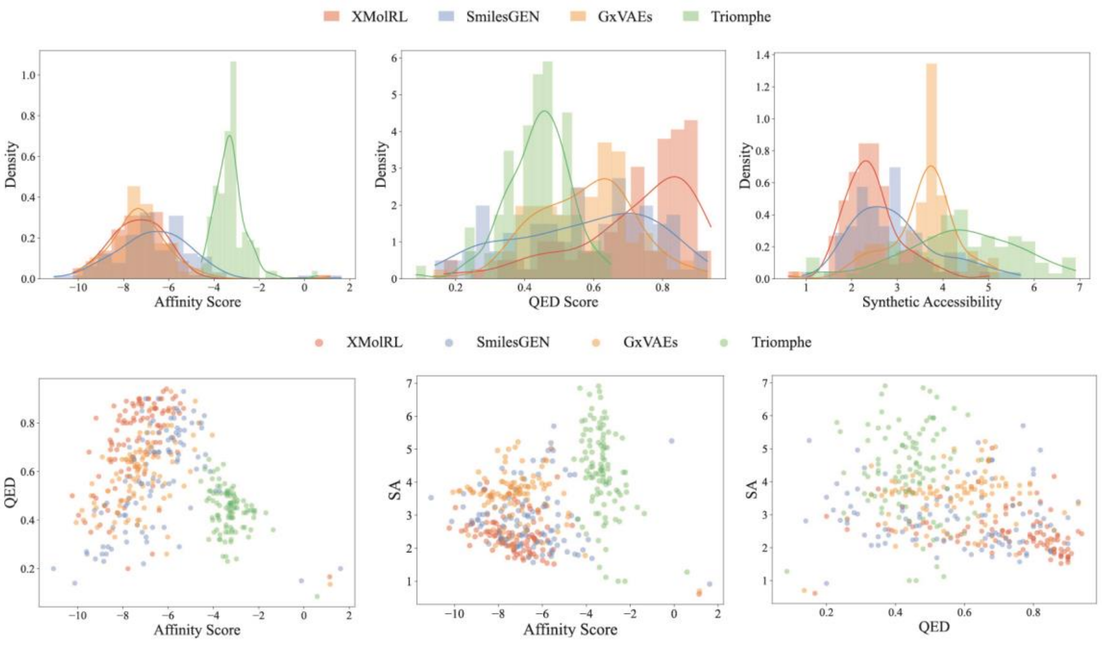

Figure S1 : Comparative Analysis of XMolRL and Phenotype-Guided Methods on Affinity, QED, and SA Metric Distributions

	XMolRL	Known Ligand	Tanimoto		XMolRL	Known Ligand	Tanimoto
AKT1	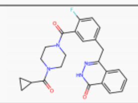	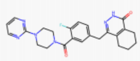	0.55	HDAC1	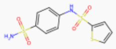	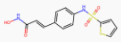	0.53
AKT2	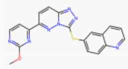	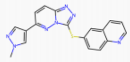	0.62	MTOR	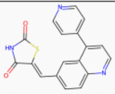	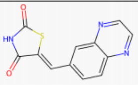	0.62
AURKB	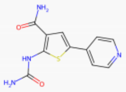	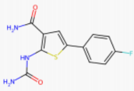	0.70	PIK3CA	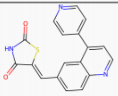	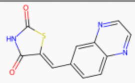	0.62
CTSK	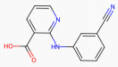	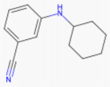	0.36	SMAD3	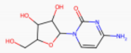	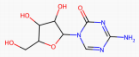	0.64
EGFR	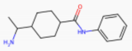	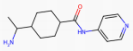	0.74	TP53	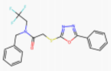	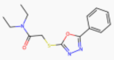	0.66

Figure S2 : Performance of Tanimoto coefficient

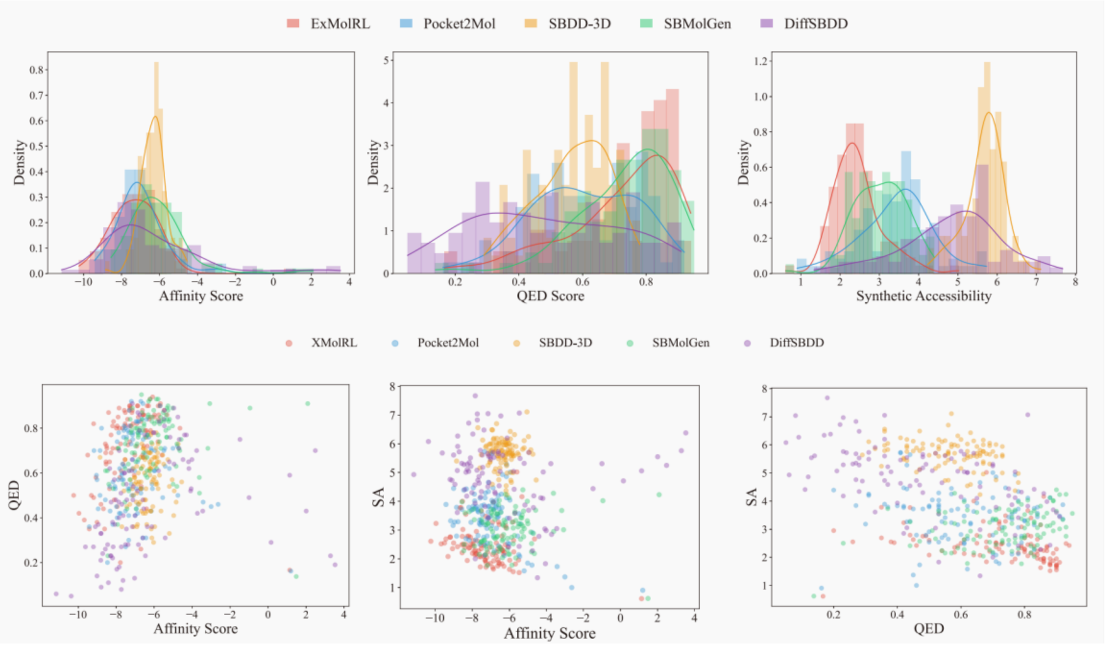

Figure S3 : Comparative Analysis of XMolRL and Target-Based Methods on Affinity, QED, and SA Metric Distributions

Table S1 : Pairwise statistical testing of predicted IC50 between XMolRL and four target-based molecular generation methods.

Target	Comparison	p-value	Cliff's delta	p-corrected	significant	stars
AKT1	XMolRL vs SBDD-3D	< 0.001	-0.533	0.000	TRUE	***
	XMolRL vs Pocket2Mol	< 0.001	-0.533	0.000	TRUE	***
	XMolRL vs SBMolGen	0.682	-0.034	1.000	FALSE	ns
	XMolRL vs DiffSBDD	0.0137	0.204	0.547	FALSE	ns
AKT2	XMolRL vs SBDD-3D	< 0.001	-0.572	0.000	TRUE	***
	XMolRL vs Pocket2Mol	0.0102	-0.211	0.406	FALSE	ns
	XMolRL vs SBMolGen	0.155	0.116	1.000	FALSE	ns
	XMolRL vs DiffSBDD	0.467	0.060	1.000	FALSE	ns
AURKB	XMolRL vs SBDD-3D	<0.001	-0.425	0.000	TRUE	***
	XMolRL vs Pocket2Mol	<0.001	-0.396	0.000	TRUE	***
	XMolRL vs SBMolGen	0.167	-0.113	1.000	FALSE	ns
	XMolRL vs DiffSBDD	0.96	0.004	1.000	FALSE	ns
CTSK	XMolRL vs SBDD-3D	<0.001	-0.410	0.000	TRUE	***
	XMolRL vs Pocket2Mol	0.005	-0.232	0.187	FALSE	ns
	XMolRL vs SBMolGen	0.588	0.045	1.000	FALSE	ns
	XMolRL vs DiffSBDD	<0.001	-0.279	0.028	TRUE	*
EGFR	XMolRL vs SBDD-3D	<0.001	-0.504	0.000	TRUE	***
	XMolRL vs Pocket2Mol	0.124	0.126	1.000	FALSE	ns
	XMolRL vs SBMolGen	0.83	-0.018	1.000	FALSE	ns
	XMolRL vs DiffSBDD	0.002	0.259	0.067	FALSE	ns
HDAC1	XMolRL vs SBDD-3D	<0.001	-0.599	0.000	TRUE	***

	XMolRL vs Pocket2Mol	<0.001	-0.604	0.000	TRUE	***
	XMolRL vs SBMolGen	0.285	-0.088	1.000	FALSE	ns
	XMolRL vs DiffSBDD	<0.001	-0.617	0.000	TRUE	***
MTOR	XMolRL vs SBDD-3D	<0.001	-0.460	0.000	TRUE	***
	XMolRL vs Pocket2Mol	<0.001	-0.323	0.003	TRUE	**
	XMolRL vs SBMolGen	0.256	-0.093	1.000	FALSE	ns
	XMolRL vs DiffSBDD	0.04	-0.168	1.000	FALSE	ns
PIK3CA	XMolRL vs SBDD-3D	<0.001	-0.358	0.001	TRUE	***
	XMolRL vs Pocket2Mol	<0.001	-0.372	0.000	TRUE	***
	XMolRL vs SBMolGen	0.478	-0.058	1.000	FALSE	ns
	XMolRL vs DiffSBDD	0.067	-0.151	1.000	FALSE	ns
SMAD3	XMolRL vs SBDD-3D	0.002	-0.250	0.091	FALSE	ns
	XMolRL vs Pocket2Mol	<0.001	-0.494	0.000	TRUE	***
	XMolRL vs SBMolGen	0.801	0.021	1.000	FALSE	ns
	XMolRL vs DiffSBDD	<0.001	-0.399	0.000	TRUE	***
TP53	XMolRL vs SBDD-3D	<0.001	-0.567	0.000	TRUE	***
	XMolRL vs Pocket2Mol	0.279	-0.082	1.000	FALSE	ns
	XMolRL vs SBMolGen	0.221	-0.100	1.000	FALSE	ns
	XMolRL vs DiffSBDD	0.72	-0.029	1.000	FALSE	ns

Note: p-value indicates statistical significance, and Cliff's delta indicates the effect size. pcorrected represents the adjusted p-value after multiple-testing correction. *, **, and *** denote $p < 0.05$, $p < 0.01$, and $p < 0.001$, respectively.

Table S2 : Performance achieved by ablated models for AKT1

Metric	XMolRL	w/o Rank	w/o QED	w/o Affinity
Uniqueness (%)	92	26	16	98
Validity (%)	98	100	100	94
Novelty (%)	91	100	100	96
Affinity (↓)	-6.461	-7.012	-6.231	-5.202
QED (↑)	0.766	0.785	0.761	0.744
SA (↓)	2.647	3.364	2.55	2.637

Table S3 : Performance achieved by ablated models for TP53

Metric	XMolRL	w/o Rank	w/o QED	w/o Affinity
Uniqueness (%)	94	28	28	85
Validity (%)	94	100	98	96
Novelty (%)	100	93	100	95
Affinity (↓)	-6.887	-6.477	-7.073	-4.665
QED (↑)	0.759	0.850	0.741	0.796
SA (↓)	2.766	2.314	2.718	2.528

Table S4 : Performance achieved by ablated models for MTOR

Metric	XMolRL	w/o Rank	w/o QED	w/o Affinity
Uniqueness (%)	98	21	61	98
Validity (%)	96	94	92	98
Novelty (%)	91	90	100	96
Affinity (↓)	-5.881	-5.762	-7.373	-4.785
QED (↑)	0.709	0.788	0.496	0.763
SA (↓)	2.600	3.335	2.673	2.711
